# Metabolomic Reprogramming Detected by ^1^H-NMR Spectroscopy in Human Thyroid Cancer Tissues

**DOI:** 10.3390/biology9060112

**Published:** 2020-05-27

**Authors:** Alessio Metere, Claire E. Graves, Mattea Chirico, Maria José Caramujo, Maria Elena Pisanu, Egidio Iorio

**Affiliations:** 1 Surgical Sciences Department, “Sapienza” University of Rome, Viale Regina Elena 261, 00161 Roma, Italy; 2 Department of Surgery, University of California, San Francisco, 1600 Divisadero St. 4th Floor, San Francisco, CA 94115, USA; claire.graves@ucsf.edu; 3 NMR Unit, Core Facilities, Istituto Superiore di Sanità, Viale Regina Elena 299, 00161 Roma, Italy; mattea.chirico@iss.it (M.C.); mariaelena.pisanu@iss.it (M.E.P.); 4 Centre for Ecology, Evolution and Environmental Changes (cE3c), Faculdade de Ciências, Universidade de Lisboa, Campo Grande, 1749-016 Lisboa, Portugal; mj.caramujo@fc.ul.pt

**Keywords:** thyroid cancer, metabolomic analyses, oxidative stress, NMR

## Abstract

Thyroid cancer cells demonstrate an increase in oxidative stress and decreased antioxidant action, but the effects of this increased oxidative stress on cell function remain unknown. We aimed to identify changes in the metabolism of thyroid cancer cells caused by oxidative stress, using proton nuclear magnetic resonance (^1^H-NMR) spectroscopy. Samples of thyroid cancer and healthy thyroid tissue were collected from patients undergoing thyroidectomy and analyzed with ^1^H-NMR spectroscopy for a wide array of metabolites. We found a significant increase in lactate content in thyroid cancer tissue compared to healthy tissue. Metabolomic analysis demonstrated significant differences between cancer tissue and healthy tissue, including an increase in aromatic amino acids, and an average decrease in citrate in thyroid cancer tissue. We hypothesize that these changes in metabolism may be due to an oxidative stress-related decrease in activity of the Krebs cycle, and a shift towards glycolysis in cancer tissue. Thus, thyroid cancer cells are able to reprogram their metabolic activity to survive in conditions of high oxidative stress and with a compromised antioxidant system. Our findings, for the first time, suggested a connection between oxidative stress and the alteration of the metabolic profile in thyroid tumors.

## 1. Introduction

The overproduction of free radicals, and consequent increase in oxidative stress, leads to irreversible cell damage, which is associated not only with cardiovascular and chronic diseases, but also with cancer [1,2,3,4,5]. In our previous studies, we used electronic paramagnetic resonance spectroscopy (EPR) to qualitatively and quantitatively evaluate the presence of oxidizing agents in the blood of patients with thyroid diseases, showing an increase in oxidative stress [6]. In addition, we have also shown in thyroid cancer a reduction of the expression of glutathione peroxidase (GPx1) and thioredoxin reductase (TrxR1), two seleno-enzymes with antioxidant action. GPx1 is expressed in cell cytosol, as well as in the mitochondria of many tissues, and it functions as an antioxidant by reducing H_2_O_2_ to water, and lipid hydroperoxides to their corresponding alcohol [7]. TrxR1 is the only enzyme known to catalyze the reduction of thioredoxins—proteins, characterized by the presence of two cysteines, that act as antioxidants by reducing oxidized proteins through cysteine thiol-disulfide exchange. In brief, the electrons are taken from NADPH via TrxR and transferred to the active site of thioredoxin, which goes on to reduce protein disulfides or other substrates [8]. Our data demonstrates the involvement of oxidative stress in thyroid tumors, consistent with previous literature, utilizing the novel method of EPR spectroscopy [9]. Compared to other methods of detecting oxidative stress, EPR spectroscopy allows us to obtain a direct measurement of the primary species, rather than its effect [10]. However, the downstream effects exerted by oxidative stress on the biochemical and metabolic pathways involved in thyroid cancer remain unknown. Nuclear magnetic resonance (NMR) represents one of the most useful techniques for metabolomic studies. NMR spectroscopic analyses allow the identification and quantification of metabolites, thus providing important information on the metabolomic pathways that may be involved in cancer. Moreover, the identification of specific molecules involved in cancer, using this refined technique, may lead to the identification of new cancer biomarkers. Lee et al. [11] have shown that NMR analyses can help to identify the metabolic pathway that can be targeted for the treatment of glioma resistant to conventional therapy, while Kim et al. [12] proposed NMR metabolomic analysis of the urine as a screening tool for early-stage colorectal cancer. Regarding the role of NMR analyses on thyroid diseases, Rezig et al. [13] showed the potential of high resolution magic angle spin (HR-MAS)-NMR-based metabolomics to improve diagnosis in thyroid lesions with indeterminate cytology after fine-needle aspiration biopsies. The purpose of this research was to identify changes in the metabolism of thyroid cancer cells caused by oxidative stress. In the present study, we performed a metabolomic analysis, using nuclear magnetic resonance (NMR) spectroscopy, to detect and evaluate the expression of metabolites present in thyroid tumor tissue compared to those found in healthy thyroid tissue. Our results allowed us to reconstruct the modifications of metabolic pathways induced by oxidative stress, showing for the first time that thyroid cancer cells undergo a sort of “metabolic reprogramming”, that make cells able to survive, even in conditions of high levels of oxidative stress.

## 2. Materials and Methods

### 2.1. Recruitment of Patients

Our research population was recruited at the Department of Surgical Sciences of the Umberto I Hospital in Rome. We enrolled 14 patients (12 women and 2 men) who underwent total thyroidectomy, aged between 26 and 64 years, and who had presented with multinodular goiter or a suspicious nodule on cytological examination after fine needle aspiration (FNA), according to the classification of the Italian Society of Pathology and Diagnostic Cytopathology [14]. FNA was performed only on thyroid nodules ≥1 cm with suspicious ultrasound characteristics, in accordance with recommendations for FNA by the American Thyroid Association (ATA) [15]. Exclusion criteria from the study included the presence of debilitating diseases (advanced stage of diabetes, immunological diseases and hematological disorders), lack of informed consent and/or authorization for the processing of personal data. All procedures involving human participants were in accordance with the 1964 Helsinki declaration and its later amendments, or comparable ethical standards, and with the ethical standards of the ethics committee of the Umberto I Hospital of Rome that approved the study (ID number: 4240). All patients provided written informed consent for the use of clinical specimens for medical research.

### 2.2. Surgical Treatment and Sampling of Thyroid Tissue for Metabolomic Analyses

Total thyroidectomy was performed based on findings from neck ultrasound, cytological diagnosis, and physical examination. After surgery, we performed a histological exam of the thyroid to determine the presence or absence of cancer. We collected 21 thyroid tissue samples, 11 taken from tumor tissue and 10 from healthy thyroid tissue. Each sample analyzed was obtained from a single biopsy of the thyroid tissue. We enrolled 9 patients affected by thyroid cancer. In one of the patients who underwent total thyroidectomy for cancer we detected 3 different foci of cancer in the same lobe, thus obtaining 3 samples of thyroid tumor tissue from this single patient. The other 8 patients presented unifocal thyroid cancer, from whom we’ve obtained the other 8 samples (*n* = 11). The healthy thyroid samples (*n* = 10) were obtained from 10 patients (5 from contralateral healthy lobe in case of cancer and 5 from thyroid in case of benign disease). Healthy thyroid samples were taken from the contralateral uninvolved thyroid lobe of patients who underwent total thyroidectomy for cancer, or from the whole thyroid, in the case of benign disease. In the case of patients with multifocal cancer, we took more than one cancer tissue sample from the same patient. The tissues collected were quickly frozen (within 30 min) and stored at −80 °C.

### 2.3. Extraction of Tissue Metabolites for Metabolomic Analysis by NMR

All samples were stored at −80 °C until metabolomic analysis with NMR spectroscopy was performed. All tissues were homogenized before the analyses. In brief, the frozen tissue pieces were weighed and placed into pre-cooled (dry ice) homogenization tubes. Ice-cold extraction solvent (10 volumes of ethanolic solution (EtOH:H_2_O, 77:23, *v*/*v*) was added to each tube in dry ice and the tissues were then homogenized by homogenizator (Ika Homogenizer T10, Sigma-Aldrich, Milan, Italy) three times over 30 s with 30 s pause intervals to ensure constant temperatures during homogenization. Then, the samples were ultra-sonicated at 20 kHz using a ultrasonic disintegrator MK2 (exponential probe, 8 µm peak-to-peak; MSE Scientific Instruments, Crawley, UK) and centrifuged at 14,000× *g* for 30 min. This method has been proven to have the same efficiency as the more common perchloric extract method, with the added benefit of a simpler experimental procedure [16]. The supernatant was then freeze-dried two times in a Savant RTV 4104 freeze dryer (Mildford, ME), and the residue resuspended in 0.7 mL of deuterated water (Sigma-Aldrich, Milan, Italy) containing 0.1 mM of 3 (trimethylsilyl)-propionic-2,2,3,3-d4 acid sodium salt (TSP) as an internal standard.

### 2.4. Metabolic Analysis by NMR Spectroscopy of the Thyroid Tissue

High-resolution NMR experiments (25 °C) were performed at 400 MHz (Bruker AVANCE spectrometer, Karlsruhe, Germany). NMR analyses on tissue extracts were carried out using a 60° flip angle pulse, preceded by 2 s presaturation for water signal suppression (interpulse delay 2 s, acquisition time 1.70 s, spectral width 12 ppm, 16,000 data points, 320 scans). Free induction decays were zero-filled to 32,000 data points and Fourier-transformed; a cubic splines model function was applied for baseline correction. Peak area correction factors were determined by experiments carried out at the equilibrium of magnetization (90° pulses, 30 s interpulse delay). The quantization of the low molecular weight metabolites was obtained from the area of the signals (obtained in conditions of non-saturation of the magnetization vector, with a repetition time between the pulses long enough to allow complete spin-lattice relaxation of the observed species), and the use of a specially implemented software (XWIN-NMR), using as standard internal TSP added to the sample. The absolute quantification of the concentration of the metabolites was assessed by measuring the areas of the signals and multiplying by suitable protection factors, using the balance of the magnetization from experiments (90° pulse, with a pulse interval of 40 s). Metabolites were identified according to our previous studies (by spiking with metabolite standards) and the databases published on the Human Metabolome Database site (http://www.hmdb.ca). The absolute quantification of metabolites was determined by comparing the integral of each metabolite to the integral of reference standard TSP, and corrected by respective proton numbers for metabolite and TSP. Metabolite quantification was expressed as nanomoles/g tissue and then converted into metabolite percentage (relative to total metabolites evaluated in each sample) to enable comparisons among the patients.

### 2.5. Statistical Analysis

All tests were applied to the metabolite percentage relative to total metabolites evaluated in each sample (nanomoles metabolite/g of tissue) in healthy versus cancer subjects (i.e., biopsy samples). Multivariate partial least squares discriminant analysis (PLS-DA) was used to explore the metabolite profiles of human thyroid tissues and evaluate the metabolic differences between the tested groups using MetaboAnalyst 4.0 available online (https://www.metaboanalyst.ca) [17,18]. The optimal number of components in the analysis was determined by cross validation for the model (leave one observation out) based on the original class assignment. Variables in the data sets were standardized (Pareto scaling) prior to PLS-DA to promote normality of data to ensure all metabolites contributed evenly to the analyses. The variable (predictor) importance in projection (VIP) statistic of Wold, which summarizes the contribution a variable makes to the model, was used to select the most important variables for projection [19]. In this study, we have considered VIP values greater than 1 to indicate important variables and from this set (7) further selected the variables with a weighted sum of absolute regression coefficient greater than 50 (5 variables, VIP > 1.3). The variable importance values reflect the contribution of each predictor in fitting the PLS model for both predictors and response while the regression coefficients represent the importance each predictor has in the prediction of just the response. All selected variables important to discriminate between healthy and cancer sample biopsies were tested by ANOVA (SPSS^®^ version 23, SPSS, Chicago, IL, USA). The *p*-values of the set of the 5 metabolites with VIP > 1.3 subjected to ANOVA, were adjusted for multiple testing using false discovery rate (FDR *q*-values) to account for multiple comparisons using a FDR online calculator that coincides with the R code of the version proposed by Benjamini and Hochberg (http://sdmproject.com/utilities/?show=FDR). To give a more accurate indication of the FDR, both *p*- and *q*-values are presented in the results when suitable. ANOVA were applied separately to each set of metabolites involved in (i) glucose/pyruvate pathways, (ii) amino acid metabolism, (iii) lipid metabolism, and (iv) energy and redox balance. The Robust Test of Equality of Means (Welch statistic) was applied instead of Fisher’s classic one-way ANOVA to L-Lysine data, which failed the test of homogeneity of variances (Levene Statistic = 10.33; *p* = 0.005) [20] using SPPS (SPSS^®^ version 23). The *p*-values from each set of metabolites subjected to ANOVA were adjusted for multiple testing using false discovery rate (FDR *q*-values) as described above.

## 3. Results

### 3.1. Relative Quantification by NMR Spectroscopy of Metabolites in Cancer and Healthy Thyroid Tissue

**Figure 1:**
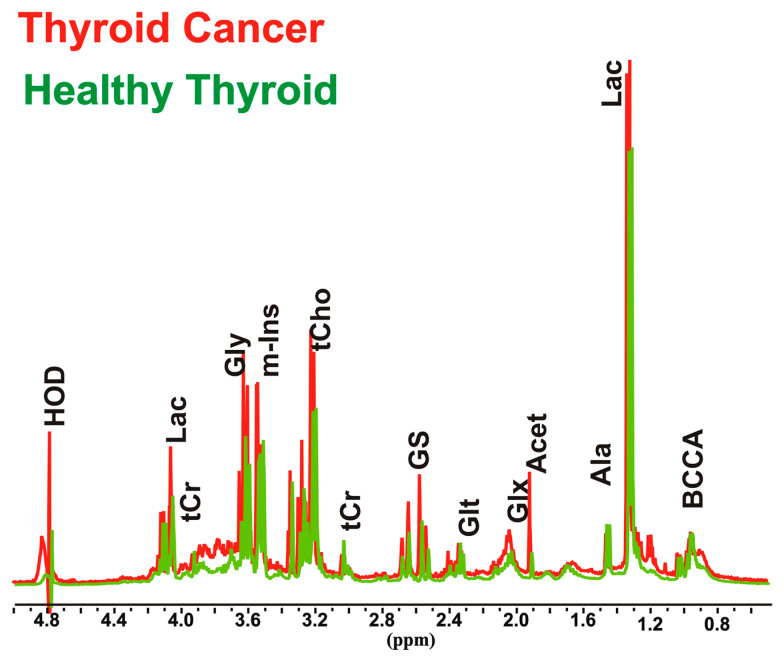
Representative partial ^1^H nuclear magnetic resonance (^1^H-NMR) spectra (9.4 T) of aqueous extracts of thyroid cancer and healthy thyroid tissues. Peak assignment: Acet, acetic acid; Ala, alanine; BCCA, branched amino acids; Gly, glycine; Glx, pool of glutamate, glutamine and glutathione; Glt, glutamate; GS, glutathione; HOD, residual H_2_O; Lac, Lactic acid; m-Ins, myo-inositol; tCr, creatine plus phosphocreatine; tCho, choline containing compounds.

Thyroid cancer tissues (*n* = 11) and healthy thyroid tissues (*n* = 10) were analyzed by NMR spectroscopy in order to clarify the biochemical and metabolic changes that occur in thyroid cancer, and to evaluate their relationship with increased oxidative stress. In order to avoid patient variability and tissue heterogeneity, the first step was to perform both absolute and relative quantification (Table S1), of 22 aqueous metabolites involved in several biochemical pathways in cancer and normal thyroid tissue spectra (Figure 1).

#### 3.1.1. Overall Metabolites in Healthy and Cancer Biopsies 

**Figure 2:**
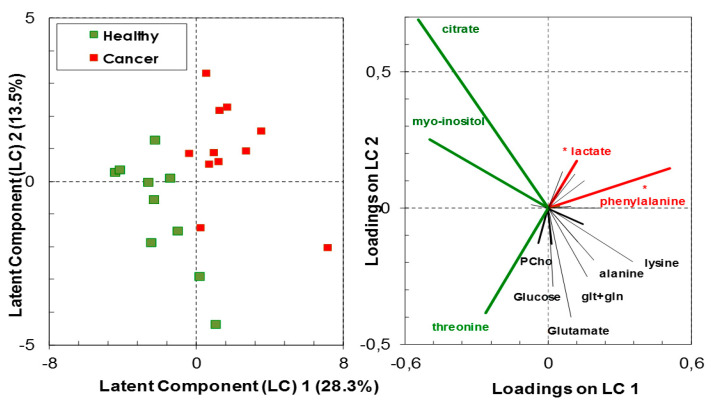
Discrimination between healthy and cancer thyroid tissue based on metabolites analyzed by high-resolution ^1^H-NMR (scores—left panel). The most important metabolites (VIP > 1.3) in the partial least squares discriminant analysis (PLS-DA) models are indicated in green or in red (loadings—right panel). Green indicates higher proportion of metabolites in healthy tissue relative to cancer tissue, while red indicates the reverse; (*) indicates metabolites contributing with more than 80% of their variance to the latent components.

High-resolution ^1^H-NMR analyses of biopsy extracts have shown a variation in the molecular proportion of various metabolites relative to the total metabolite content between healthy and cancer tissues. Class membership of healthy vs. cancer biopsies could not be accurately predicted by the linear combination of the assessed metabolites with a predictive ability (Q2) of 0.49 (predicted R2 = 0.83) with the three components of the PLS-DA model containing 55.4% of data variance (Figure 2 and Figure S1). Nevertheless, the analysis has indicative value with the following significant metabolites in group classification, having both high predictor importance in projection (VIP > 1.3) and correlation coefficient above 50%: L-phenylalanine and lactate, which had positive loadings on the latent component 1 (LC1); citrate, myo-inositol and threonine, which had negative loadings on LC1. The first group of metabolites were in significantly higher concentrations in the cancer thyroid tissue than in the healthy tissue (F_1,19_ = 13.94, *p* = 0.001, *q* = 0.005 for L-phenylalanine; F_1,19_ = 5.348, *p* = 0.032, *q* = 0.08 for lactate), while the second group of metabolites were in higher, albeit not significantly, concentration in the healthy thyroid tissue (ANOVA *p* > 0.05) (Figure 2).

#### 3.1.2. Metabolites in Biochemical Pathways

**Figure 3:**
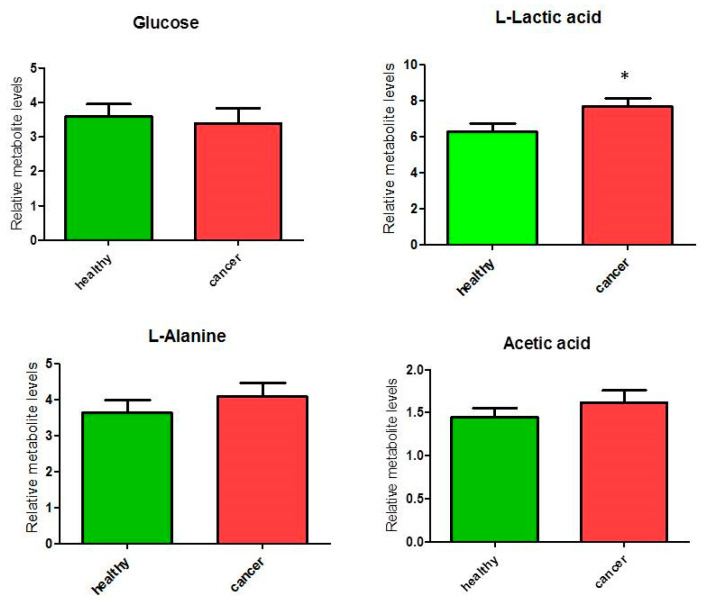
Percentage of metabolites (nmoles of metabolite relative to total nmoles of metabolites per g of tissue) involved in the glucose/pyruvate pathways in thyroid cancer tissue (*n* = 11) and healthy thyroid tissues (*n* = 10). Values are mean ± SD; (*) indicates ANOVA F_1,19_ = 5.39; *p* = 0.032 and FDR *q* = 0.128.

We analyzed metabolites involved in glucose/pyruvate metabolism, and a significant (*p* < 0.05) increase of 22% was observed only for lactate content (ANOVA F_1,19_ = 5.39; *p* = 0.032, although FDR *q* = 0.128) in cancer tissues compared to healthy tissues in the glucose/pyruvate pathways (Figure 3). The levels of glucose did not differ between cancer and healthy tissues, while we observed an average increase (but not significant) of alanine and acetic acid linked to pyruvate metabolism. These observations suggest an increase of glycolytic activity in thyroid cancer tissues.

**Figure 4:**
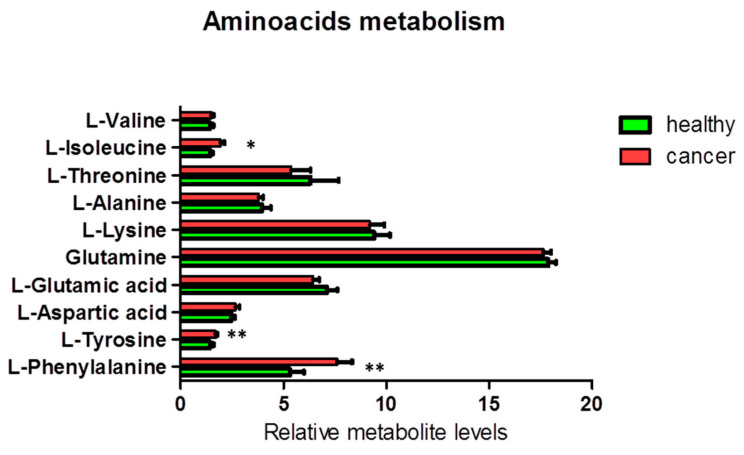
Percentage of metabolites (nmoles of metabolite relative to total nmoles of metabolites per g of tissue) involved in the amino acid metabolism in thyroid cancer tissue (*n* = 11) and healthy thyroid tissues (*n* = 10). Values are mean ± SD; (*) indicates ANOVA F values with *p* < 0.05, and (**) indicates *q* < 0.05 when FDR correction is applied for multiple comparisons.

Additionally, we found a significant increase in the levels of the aromatic amino acids tyrosine (ANOVA F_1,18_ = 9.38; *p* = 0.007; FDR *q* = 0.035) and phenylalanine (ANOVA F_1,19_ = 13.94; *p* = 0.001; FDR *q* = 0.010) and a less significant increase in the level of the branched-chain amino acid (BCAA) isoleucine (ANOVA F_1,19_ = 6.60; *p* = 0.019; FDR *q* = 0.063) in cancer tissues, as compared to the corresponding healthy tissues (Figure 4). Other NMR-detectable amino acids were not significantly different between cancer and healthy tissues.

**Figure 5:**
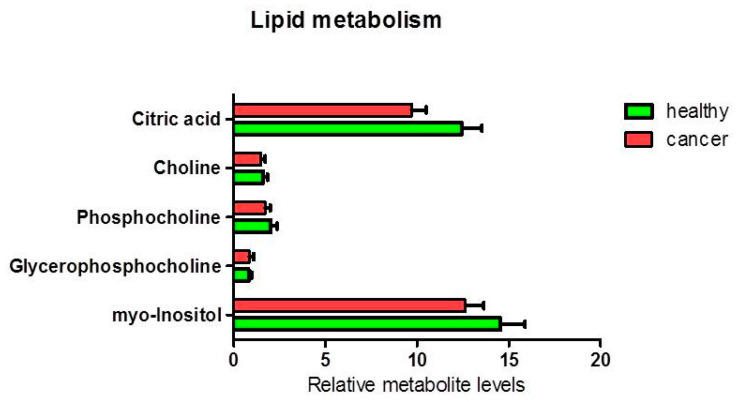
Percentage of metabolites (nmoles of metabolite relative to total nmoles of metabolites per g of tissue) involved in lipid metabolism in thyroid cancer tissue (*n* = 11) and healthy thyroid tissues (*n* = 10). The tendencies of variation of metabolites between healthy and cancer tissues were not statistically significant (highest ANOVA F_1,17_ = 4.05; *p* = 0.060 for citrate).

**Figure 6:**
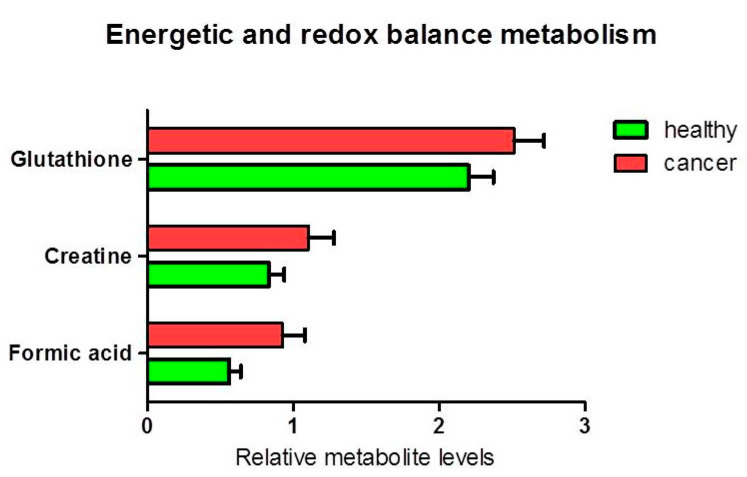
Percentage of metabolites (nmoles of metabolite relative to total nmoles of metabolites per g of tissue) involved in energy and redox balance in thyroid cancer tissue (*n* = 11) and healthy thyroid tissues (*n* = 10). The tendencies of variation of metabolite % between healthy and cancer tissues were not statistically significant (ANOVA *p* > 0.05).

The analysis of other metabolites involved in lipid metabolism (Figure 5) and cellular energy and redox balance metabolism (Figure 6), did not show any statistically significant difference between the levels of polar phosphatidylcholine and phosphatidylinositol in healthy relative to cancer tissues. Nevertheless, a slight decrease of the citrate content was observed in cancer tissues (ANOVA F_1,17_ = 4.05; *p* = 0.060). An average decrease in citrate and increase in acetic acid in cancer tissues, could be compatible with de novo lipid synthesis and histone acetylation. Furthermore, we found a statistically significant increase in formic acid in cancer tissue compared to healthy tissue (*p* = 0.037). There was an average increase in the pool of glutathione (*p* = 0.087) and creatine (plus phosphocreatine) not statistically significant.

## 4. Discussion

**Figure 7:**
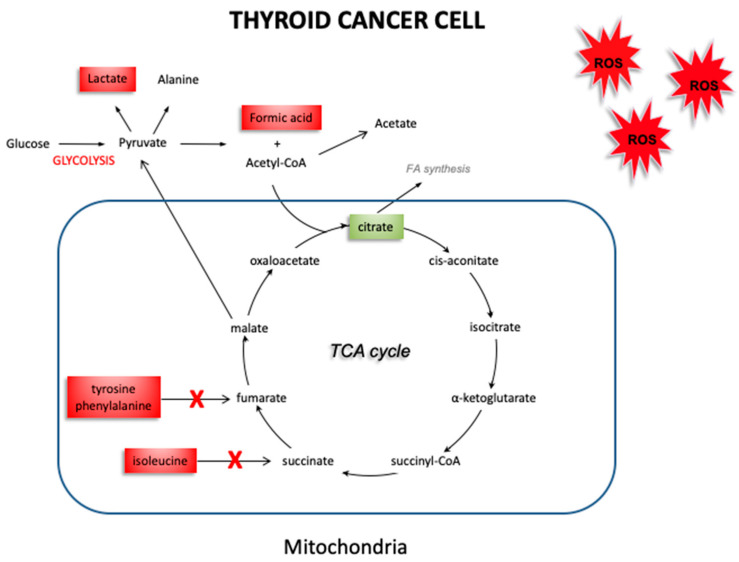
Schematic picture of altered metabolism in thyroid cancer. The reduction observed in the intermediates produced during the Krebs cycle (citrate), along with an accumulation of tyrosine, phenylalanine, isoleucine, acetate and formic acid may be attributable to a decrease in activity of the Krebs cycle, and a shift towards glycolysis in cancer tissue. (The red and green boxes indicate an increase or a decrease of the metabolites’ concentration, respectively).

Cancer cells tend to have increased production of free radicals, as well as a compromised antioxidant defense system that is no longer able to counteract the overproduction of free radicals [21,22]. This leads to an increase in oxidative stress, with the impairment of physiological activities such as cell growth and mitosis [23]. Our group has previously reported an imbalance in the oxidant/antioxidant system in thyroid cancer. In fact, the decrease in the antioxidant enzymes GPx1 and TrxR1 found in thyroid cancer suggest that the antioxidant systems of thyroid cancer cells are not able to adequately counteract the effect of free radicals. The use of EPR spectroscopy [24] associated to the CPH spin trap, helped us to detect an increase in oxidative stress in thyroid cancer, by obtaining a direct measure of the responsible free radicals. These results support different hypotheses. On the one hand, cancer cells ability to protect themselves from oxidative stress may be impaired due to a quantitative and qualitative deficit of antioxidant systems. On the other hand, this deficit could be, instead, the consequence of an increase in consumption of the antioxidant cellular reserve, due to an overproduction of free radicals. However, it is still unclear what effects this oxidative stress could have in thyroid cancer. NMR spectroscopy allowed us to explore the changes in the metabolism of thyroid cancer tissues, which may be related to an increase in oxidative stress. Figure 6 shows a schematic picture obtained by our results, which highlights the expression of the various metabolites in healthy thyroid vs. thyroid cancer tissue. In thyroid cancer tissues, we observed a metabolic “reprogramming” which includes changes in the metabolites involved in mitochondrial activities (citrate), and in glucose/pyruvate metabolism. The difference in citrate levels, as well as the increase in acetate content, support lipid biogenesis in thyroid cancer tissues. Mitochondria tightly regulate cellular processes involved in energy production and homeostasis, producing ATP and reactive oxygen species (ROS). The ROS content could dramatically increase when the respiratory chain is dysfunctional and alter mitochondrial activity, leading to enhanced glycolysis. Our quantitative results have shown an average increase in lactate levels in thyroid cancer tissues, suggesting a metabolic shift towards glycolysis in these cancer tissues. Taking into account that changes in the redox status affect the oxidative and energetic metabolism, we decided to verify the role of oxidative stress in thyroid cancer cell metabolism. Our findings demonstrate an increase in glycolytic activity, associated with the increase of ROS levels in cancer tissues we detected in our previous study [9]. These data were confirmed by the increase in lactate concentration in thyroid cancer tissues. In our previous study, we showed that the alteration in the metabolic profile (switch towards glycolysis) is due to ROS, since it was reverted when cells were cultured in the presence of antioxidant N-acetylcysteine [25]. Therefore, we hypothesize that ROS are also the determinant of this metabolic alteration. Furthermore, as reported by Tauffenberger et al., lactate may promote oxidative stress resistance through ROS signaling, as seen in neuroblastoma and in the nematode *Caenorhabditis elegans* [26]. High content of lactate and choline, and low levels of citrate, glutamine, and glutamate were reported by Ryoo et al. [27] in malignant thyroid nodules, and were suggested as the discriminative markers for determining the preoperative metabolomic profile of thyroid nodules. High levels of lactate have also been found in several other neoplasias, including breast, glioma, and head and neck cancers [28]. Elevated levels of lactate result from an increase in the glycolytic pathway following hypoxia or ischemia in tumor tissues. Lactate is known as an important factor in terms of cancer cell mobility and immune suppression [29]. Several studies have reported a significant alteration of lipid profiles in thyroid carcinoma, compared to adjacent healthy tissue. These studies have shown different concentrations of fatty acids at the level of phosphatidylcholine [30,31,32] and high levels of choline in tumor tissues [27,33]. Furthermore, it has also been observed that malignant thyroid lesions have lower citrate levels than benign ones [34,35], as also demonstrated in our results. Studies of in-vitro cultured cells suggest that oncogenic activation of BRAF (often mutated in thyroid cancer) inhibits OXPHOS in mitochondria and promotes aerobic glycolysis to sustain cancer cell growth [36]. Our findings, for the first time, hypothesize a connection between oxidative stress and the alteration of the metabolic profile in thyroid tumors. We observed by EPR spectroscopy a significant increase in the ROS content of approximately double in thyroid cancer samples, associated with a reduction in the levels of the antioxidant enzymes GPx1 and TrxR1, and now suggest a kind of metabolic “reprogramming” in thyroid cancer cells, assessed through NMR spectroscopy. Specifically, the results obtained with NMR spectroscopy suggest reduced activity of the Krebs cycle. The reduction in citrate, produced during the Krebs cycle, may be attributed to a decrease in oxidative metabolism in neoplastic cells, consistent with an accumulation of precursors such as acetate. Furthermore, impairment of oxidative metabolism would also explain the reduction in the formation of acetyl CoA, with the resultant accumulation of its precursor, acetate (Figure 7). 

Our findings also show an increase in total glutathione—likely due to enhanced oxidant environment [ROS]. The metabolomic analysis performed (and the type of instrumentation used) did not allow us to discriminate between reduced or oxidized glutathione, only showing an increase in total glutathione. Our findings, of course, only partially clarify the complex mechanisms underlying thyroid cancer. Other studies are needed to better understand the role of other metabolites and enzymes involved in thyroid cancer, as well as the mechanisms involved in the overproduction of free radicals. However, the data is quite clear that thyroid cancer cells are able to reprogram their metabolic activity to survive in conditions of high oxidative stress and with a compromised antioxidant system. A recent study by Yanyun Li et al [37] on 16 patients reported that the metabolome of papillary thyroid carcinoma (PTC) is characterized by increased glycolysis and inhibited tricarboxylic acid cycle, with increased oncogenic amino acids (isoleucine) as well as abnormal choline and lipid metabolism. An increase in isoleucine levels, together with high levels of other branched-chain amino acids are often associated with the risk of PTC [38]. Other studies have suggested metabolites involved in different pathways such as alanine, methionine, acetone, glutamate, glycine, lactate, tyrosine, phenylalanine and hypoxanthine as potential biomarkers common to all thyroid lesions [39]. Finally, analyses of intact samples by HR-MAS technology showed an increase of phenylalanine, taurine, and lactate and a decrease of choline and choline derivatives, myo- and scyllo-inositol in malignant thyroid tumors compared to benign tissue [40]. Chronic administration of L-tyrosine results in a marked decrease in the activity of citrate synthase and succinate dehydrogenase activities in hippocampus and striatum, and that antioxidants administration (N-acetylcysteine, and deferoxamine) could prevent this inhibition in hippocampus and striatum [41].

## 5. Conclusions

Thyroid cancer cells show evidence of increased oxidative stress and impaired antioxidant defenses. In this study, using NMR spectroscopy to explore differences in the metabolites of thyroid cancer tissue compared to healthy tissue, we demonstrate evidence of metabolic “reprogramming” in cancer cells, with changes in the metabolites involved in mitochondrial activity and in glucose/pyruvate metabolism. Our findings suggest that thyroid cancer cells have metabolic plasticity that allows them to survive in extreme environmental conditions, such as those with high oxidative stress levels. The metabolic pathways involved in this adaptation may provide additional targets for cancer-fighting therapeutic agents. Lactate, for example, may be considered not only as a cancer marker but also as an oncometabolite potentially capable of increasing cellular resistance to harmful stimuli. The metabolic pathways involved in lactate synthesis may represent a novel molecular target to counteract thyroid cancer.

## Supplementary Materials

The following are available online at https://www.mdpi.com/2079-7737/9/6/112/s1, Figure S1: The variable importance in projection (VIP) for the analyzed metabolites in the PLS-DA model, Table S1: Absolute and relative quantification of metabolites detected by NMR in thyroid cancer and healthy thyroid tissues.
