# Supplementary material for: Metabolomic Reprogramming Detected by 1H-NMR Spectroscopy in Human Thyroid Cancer Tissues
Source: Biology (Basel). 2020 May 27;9(6):112. doi: 10.3390/biology9060112 (PMC7345942; doi:10.3390/biology9060112)
Supplement: Supplementary file 1 [file biology-09-00112-s001.zip › Table S1Absolute and relative quantification of metabolites detected by NMR in thyroid cancer and healthy thyroid tissues..docx]

| **Metabolites** | **Absolute Quantification (nmoles/g of tissue)** | | | | | | **Relative Quantification (% [metabolite]/[all metabolites])** | | | | | | |
| --- | --- | --- | --- | --- | --- | --- | --- | --- | --- | --- | --- | --- | --- |
|  | **healthy** | | | **cancer** | | | **healthy** | | | **cancer** | | |  |
|  | median | range (min-max) | | median | range (min-max) | | median | range (min-max) | | median | range (min-max) | |  |
| Formic acid | 37,13 | 17,18 | 865,44 | 211,00 | 13,57 | 2822,75 | 0,64 | 0,17 | 0,92 | 0,90 | 0,24 | 1,98 |  |
| L-Phenylalanine | 1078,81 | 112,37 | 5286,78 | 1371,19 | 120,21 | 19052,91 | 4,81 | 2,23 | 6,82 | 7,75 | 5,57 | 13,34 |  |
| L-Tyrosine | 271,72 | 32,97 | 1465,11 | 327,13 | 29,50 | 2286,66 | 1,27 | 0,92 | 1,78 | 1,67 | 1,09 | 2,88 |  |
| D-Glucose | 831,56 | 64,57 | 6299,92 | 1484,18 | 59,48 | 7261,90 | 3,83 | 1,51 | 4,89 | 3,10 | 1,22 | 5,79 |  |
| L-Lactic acid | 1157,31 | 155,87 | 7615,39 | 1874,41 | 94,57 | 8938,99 | 6,71 | 4,03 | 8,40 | 7,10 | 5,73 | 10,96 |  |
| myo-Inositol | 3668,40 | 362,15 | 15233,84 | 2672,90 | 167,63 | 14193,42 | 15,22 | 9,44 | 21,58 | 12,98 | 7,24 | 17,04 |  |
| Glycerophosphocholine | 233,08 | 15,97 | 946,95 | 172,39 | 8,80 | 843,33 | 0,72 | 0,20 | 2,19 | 0,65 | 0,21 | 2,69 |  |
| Phosphocholine | 608,62 | 56,51 | 1852,54 | 397,25 | 31,15 | 2067,10 | 1,95 | 0,92 | 5,00 | 1,74 | 0,51 | 3,13 |  |
| Choline | 361,19 | 39,90 | 3042,25 | 393,03 | 12,25 | 1459,85 | 1,46 | 0,27 | 2,89 | 1,36 | 0,17 | 2,56 |  |
| Creatine | 208,90 | 13,53 | 746,48 | 186,18 | 14,75 | 1205,89 | 0,70 | 0,46 | 1,59 | 0,94 | 0,47 | 2,16 |  |
| L-Aspartic acid | 480,00 | 51,09 | 3041,93 | 1246,95 | 57,07 | 4130,88 | 2,39 | 1,75 | 3,25 | 2,53 | 1,56 | 3,46 |  |
| Citric acid | 2280,99 | 377,94 | 14075,66 | 3450,90 | 125,70 | 12073,33 | 11,00 | 6,74 | 17,31 | 8,79 | 6,97 | 13,86 |  |
| Glutathione | 486,06 | 39,87 | 3106,72 | 682,71 | 37,86 | 4260,35 | 2,00 | 1,44 | 3,10 | 2,84 | 1,60 | 3,32 |  |
| Succinic acid | 97,95 | 14,68 | 428,89 | 155,38 | 11,28 | 907,29 | 0,49 | 0,27 | 0,78 | 0,60 | 0,41 | 3,39 |  |
| L-Glutamic acid | 1564,56 | 136,66 | 7814,71 | 1474,21 | 97,51 | 6448,15 | 6,64 | 4,51 | 10,76 | 6,50 | 4,52 | 8,11 |  |
| Glutamine | 3740,72 | 428,86 | 21843,08 | 3529,63 | 265,56 | 25313,89 | 17,41 | 15,51 | 19,88 | 17,81 | 15,60 | 19,91 |  |
| Acetic acid | 264,40 | 46,24 | 1562,19 | 306,98 | 8,43 | 2779,64 | 1,50 | 0,91 | 2,01 | 1,68 | 0,63 | 2,19 |  |
| L-Lysine | 1795,80 | 169,66 | 12764,18 | 1622,59 | 96,90 | 12584,03 | 8,73 | 7,37 | 9,90 | 8,81 | 7,27 | 16,83 |  |
| L-Alanine | 897,31 | 60,10 | 4521,46 | 932,19 | 55,97 | 4407,59 | 3,59 | 1,57 | 5,27 | 4,10 | 2,52 | 7,06 |  |
| L-Threonine | 1596,23 | 57,11 | 16911,80 | 5694,68 | 333,06 | 6396,05 | 6,61 | 1,18 | 13,12 | 3,99 | 2,36 | 8,26 |  |
| L-Isoleucine | 320,38 | 28,29 | 1361,77 | 359,15 | 21,33 | 2640,24 | 1,59 | 0,47 | 1,92 | 1,80 | 1,22 | 3,47 |  |
| L-Valine | 368,81 | 16,68 | 1455,73 | 657,01 | 10,42 | 2579,65 | 1,28 | 0,72 | 2,21 | 1,65 | 0,78 | 2,69 |  |

Supplemental tabel 1 Absolute and relative quantification of metabolites detected by NMR in thyroid cancer and healthy thyroid tissues.
