# Supplementary figures and images for: Metabolomic Reprogramming Detected by 1H-NMR Spectroscopy in Human Thyroid Cancer Tissues
Source: Biology (Basel). 2020 May 27;9(6):112. doi: 10.3390/biology9060112 (PMC7345942; doi:10.3390/biology9060112)

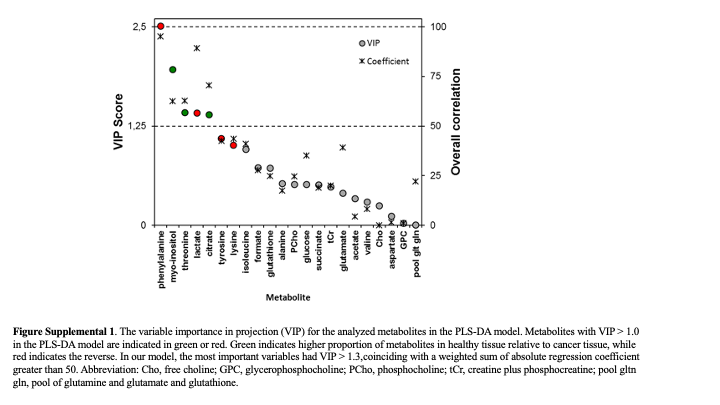

Supplement: Supplementary file 1 [file biology-09-00112-s001.zip › Figure S1 The variable importance in projection (VIP) for the analyzed metabolites in the PLS-DA model.tiff]
